# Functional Expression of Thyroid-Stimulating Hormone Receptor on Nano-Sized Bacterial Magnetic Particles in *Magnetospirillum magneticum* AMB-1

**DOI:** 10.3390/ijms140714426

**Published:** 2013-07-11

**Authors:** Yasuhiro Sugamata, Ryo Uchiyama, Toru Honda, Tsuyoshi Tanaka, Tadashi Matsunaga, Tomoko Yoshino

**Affiliations:** Division of Biotechnology and Life Science, Institute of Engineering, Tokyo University of Agriculture and Technology, 2-24-16, Naka-cho, Koganei, Tokyo 184-8588, Japan; E-Mails: 50011702104@st.tuat.ac.jp (Y.S.); ucchi.time-to-say-goodbye@ymail.plala.or.jp (R.U.); 50012831207@st.tuat.ac.jp (T.H.); tsuyo@cc.tuat.ac.jp (T.T.); tmatsuna@cc.tuat.ac.jp (T.M.)

**Keywords:** thyroid-stimulating hormone receptor, *Magnetospirillum magneticum* AMB-1, autoantibody, bacterial magnetic particles, tetracycline-inducible expression system

## Abstract

The measurement of autoantibodies to thyroid-stimulating hormone receptor (TSHR) is important for the diagnosis of autoimmune thyroid disease such as Graves’ disease (GD). Although TSHR from porcine thyroid membrane is commonly used for the measurement of TSHR autoantibodies (TRAb), recombinant human TSHR (hTSHR) remains ideal in terms of stable supply and species identity. Here we set out to express recombinant hTSHR on the lipid-bilayer surface of magnetic nanoparticles from a magnetotactic bacterium, *Magnetospirillum magneticum* AMB-1. Using a tetracycline-inducible expression system, we successfully overexpressed functional hTSHR on bacterial magnetic particles (BacMPs) in AMB-1 via an anchor protein specific for BacMPs. The overexpressed hTSHR was membrane integrated and possessed both ligand and autoantibody binding activity. Our data suggest that hTSHR-displayed BacMPs have potential as novel tools for ligand-receptor interaction analysis or for TRAb immunoassay in GD patients.

## 1. Introduction

Thyroid-stimulating hormone receptor (TSHR) belongs to the subfamily of rhodopsin-like members of the G-protein coupled receptor (GPCR) superfamily, and plays a central role in thyroid hormone production and regulation [1]. The stimulation of autoantibodies to TSHR (TRAbs) is known to be associated with hyperthyroidism in Graves’ disease (GD), and measurement of TRAbs is important for diagnosis of GD [2,3].

Currently available immunoassays for measuring TRAb are competitive radioimmunoassay using I^125^-labelled TSH or enzyme-linked immunosorbent assay (ELISA) using biotin-labeled TSH (TSH-biotin) [4,5]. More recently, a biotin-labeled human monoclonal thyroid stimulating antibody, M22, has been used for TRAb ELISA instead of TSH-biotin [6]. In these assays, preparation of functional TSHR protein is a critical step. Given that TSHR, like other typical GPCRs, is notoriously difficult to overexpress in a soluble form, detergent-extracted porcine thyroid membrane is generally used as a source of TSHR instead of human TSHR (hTSHR) in current TRAb immunoassays. However, the use of thyroid membrane extract carries with it the potential for lot-to-lot inconsistency, and differences in species may influence the detection of autoantibodies to hTSHR [7]. To avoid these possible risks, the development of TRAb assay using recombinant hTSHR is desirable.

*Magnetospirillum magneticum* AMB-1 is a gram-negative, facultative anaerobic bacterium that is known to produce bacterial magnetic particles (BacMPs) which form a magnetosome chain in the cytoplasm under anaerobic conditions. BacMPs, which are typically 50–100 nm in size, consist of magnetite (Fe_3_O_4_) surrounded by a lipid bilayer membrane, and exhibit strong ferrimagnetism. Several membrane-integrated or tightly bound proteins are known to be abundant on the surface of BacMPs [8]. Using these characteristics, we have succeeded to date in functionally displaying soluble proteins on BacMPs by gene fusion techniques, using either MagA, Mms16, or Mms13 as an anchor molecule, with applications in purposes such as immunoassay, enzyme reaction, ligand-receptor interaction or cell separation [9–12]. The main advantage of the BacMP-based expression system is that the protein of interest is easily and directly isolated using a magnet. We recently applied these techniques to overexpress transmembrane proteins such as D1 dopamine receptor, a member of the GPCR family, and a truncated form of CD81, a tetraspanin receptor for Hepatitis C Virus, and demonstrated ligand-binding activity [10,13]. However, applications of transmembrane proteins, especially of GPCRs, are currently limited.

Here we describe the successful expression of Mms13-anchored full-length hTSHR in *M. Magneticum* AMB-1 using a tetracycline-inducible expression system, and demonstrations of its ligand and autoantibody-binding activity. This study raises the possibility of applications using hTHSR-displayed BacMPs for the analysis of ligand or autoantibody-receptor interaction, or for automated TRAb immunoassay.

## 2. Results

### 2.1. Growth of hTSHR Transformants in a Tetracycline-Inducible Expression System

We have previously observed extremely low expression of Mms13-hTSHR on BacMPs due to growth inhibition when constitutively overexpressed in *M. Magneticum* AMB-1 (data not shown). Accordingly, we investigated the use of a tetracycline-inducible expression system [13].

**Figure 1:**
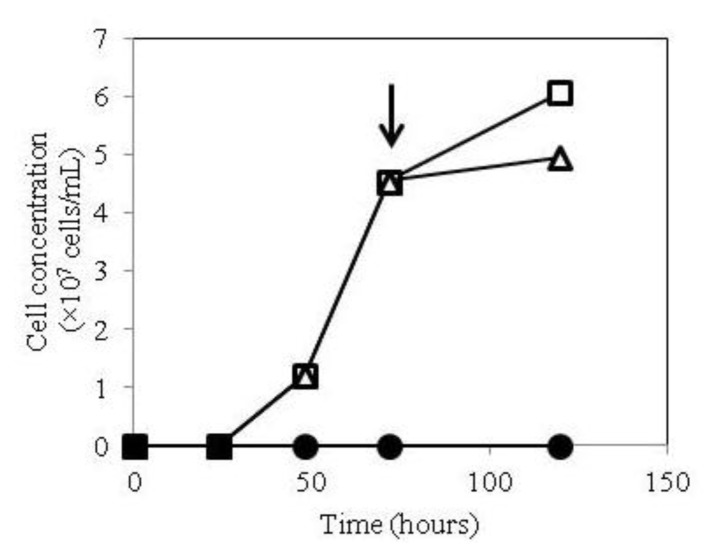
Growth curves of the AMB-1 transformant of pUMtOR13TSHR. The transformant was grown in magnetic spirillum growth medium (MSGM) with or without ATc. ATc (500 ng/mL) was added to the medium at the time of inoculation (filled circle) or at mid-log phase, indicated by solid arrow (open triangles). Open squares show growth curves in the absence of ATc.

*M. Magneticum* AMB-1 transformants harboring pUMtOR13TSHR (see Experimental section) were grown in magnetic spirillum growth medium (MSGM) with or without addition of anhydrotetracycline (ATc). When ATc was added at the start of inoculation, no growth of the TSHR transformant was observed, which was consistent with the previous result of constitutive expression (Figure 1). Likewise, the hTSHR transformant, but not wild-type AMB-1, underwent significant growth inhibition after the addition of ATc at mid-log phase (Figure 1). These results indicate that expression of Mms13-hTSHR is toxic to AMB-1, and that inducible expression is necessary.

### 2.2. Isolation of hTSHR-Displaying BacMPs

**Figure 2:**
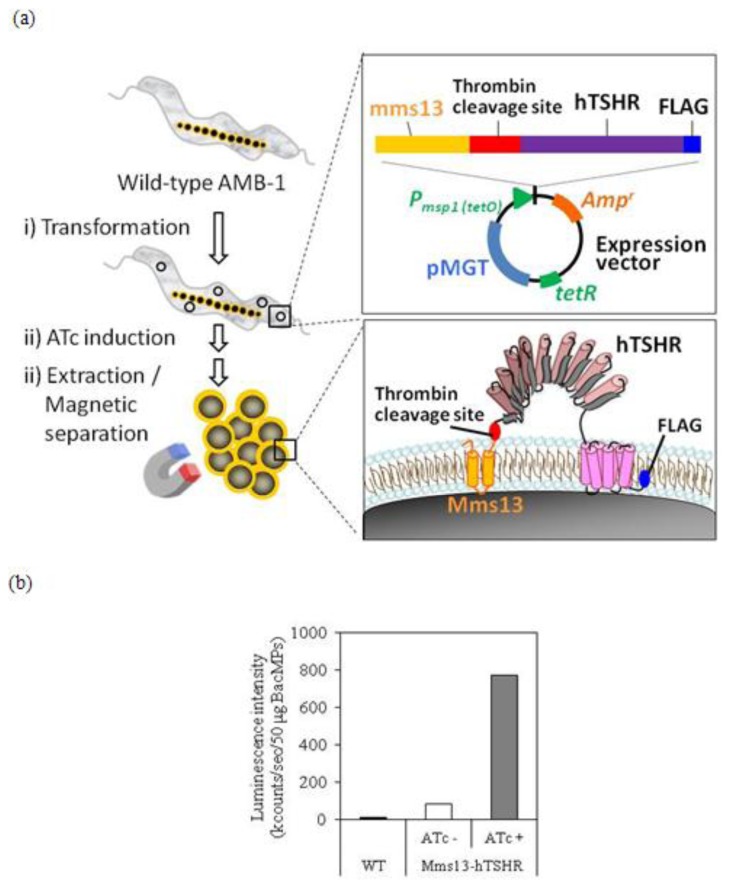
Confirmation of hTSHR expression on BacMPs. (**a**) Schematic diagram for preparation of BacMPs displaying hTSHR. Plasmids pUMtOR13TSHR was introduced in the wild-type AMB-1 for expression on BacMPs (step i). After ATc induction (step ii), hTSHR-BacMPs were magnetically separated and purified by repeated washing (step iii); (**b**) ATc-induced expression of hTSHR on BacMPs. Immunoassay on BacMPs was performed after 72-h induction (shown by an arrowhead) with ATc at mid-log phase. hTSHR expression on BacMPs was detected using anti-human TSHR monoclonal antibody. WT, wild-type AMB-1; ATc-, AMB-1 transformant of pUMtOR13TSHR without ATc induction; ATc+, AMB-1 transformant with ATc induction at 500 ng/mL.

Figure 2a shows the procedure for isolation of hTSHR-displaying BacMPs. 6.5 mg of BacMPs were isolated from a 5 L culture of AMB-1 transformants of Mms13-hTSHR after induction with ATc. Inducible expression of the Mms13-hTSHR fusion protein on BacMPs was evaluated by ELISA using anti-hTSHR antibody. As shown in Figure 2b, low-level expression of Mms13-hTSHR was observed without induction of ATc. On the other hand, an approximately 9-fold increase in the expression of Mms13-hTSHR was detected on BacMPs in transformants after ATc induction (Figure 2b), demonstrating that inducible expression system is effective for hTSHR expression on BacMPs.

### 2.3. Detection of Mms13-hTSHR Fusion Protein by Immunoblotting

**Figure 3:**
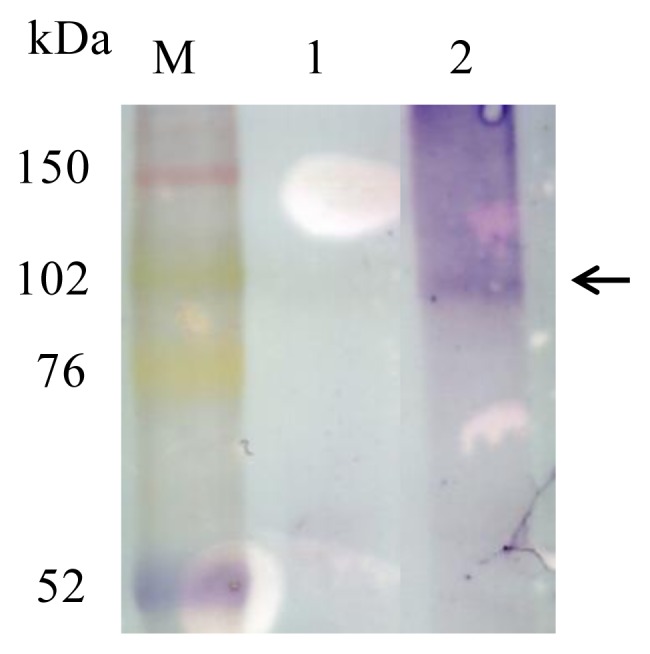
Expression analysis of hTSHR on BacMPs by Western blotting. M, Marker; Lane 1, WT; Lane 2, AMB-1 transformant of pUMtOR13TSHR. Arrow indicates a protein band corresponding to Mms13-hTSHR (~100 kDa).

To identify the Mms13-hTSHR fusion protein on BacMPs, BacMPs extracted from the transformant were subjected to immunoblotting. We used a monoclonal antibody to the *C*-terminal FLAG tag because we wanted to confirm that the Mms13-hTSHR fusion protein was fully translated and found at the expected size. A band was observed corresponding to ~100 kDa for Mms13-hTSHR, indicating the fusion protein was expressed on BacMPs at the correct size (Figure 3). However, we also detected a broad smeared band of larger size, which may be aggregates of Mms13-hTSHR caused by heating during sample preparation. From our experiences, transmembrane proteins on BacMPs are highly prone to aggregate during heating step for sample preparation, which is necessary to release Mms13 fusion protein from BacMPs completely. Similar observation is also reported elsewhere [14].

### 2.4. Membrane Integration Analysis

**Figure 4:**
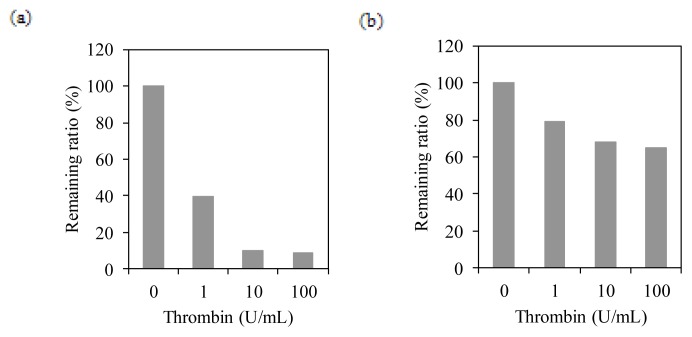
Thrombin cleavage assay. Remaining ratio of GFP (**a**) or hTSHR (**b**) fusion proteins on BacMPs after thrombin cleavage. Remaining ratio: 100% = signal at 0 unit/mL thrombin; 0% = signal of non-specific binding of AP-conjugated antibody.

We next investigated whether the transmembrane region of hTSHR is integrated into the lipid bilayer membrane of BacMPs. BacMPs were extracted from the AMB-1 transformant expressing Mms13-hTSHR or Mms13-green fluorescent protein (GFP) fusion protein (Mms13-GFP, as a control) and treated with thrombin, then analyzed for each remaining protein on BacMPs using antibodies to individual proteins. At an effective concentration of thrombin (≥10 U/mL), the majority of the GFP on BacMPs was cleaved at the thrombin site between Mms13 and GFP (Figure 4a). In contrast, >50% of hTSHR remained on BacMPs even in the presence of 100 U/mL of thrombin (Figure 4b). These results suggest that a proportion of hTSHR is integrated in the lipid bilayer membrane of BacMPs.

### 2.5. Binding of TSH to hTSHR on BacMPs

**Figure 5:**
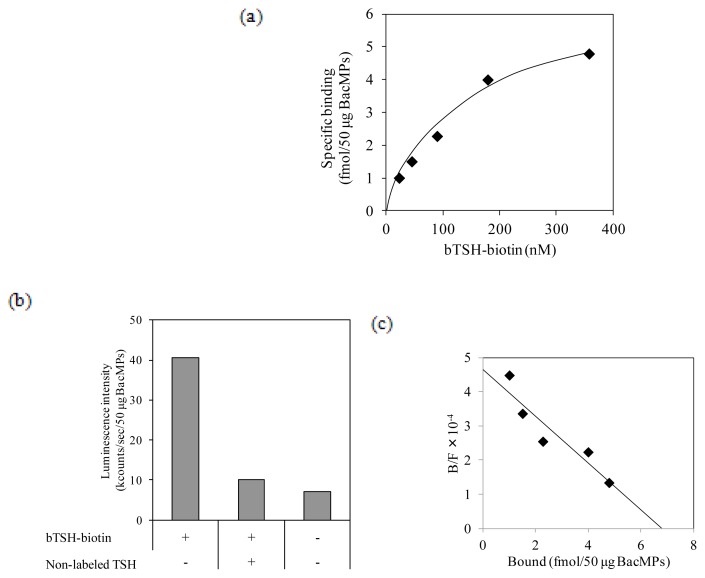
Binding of bTSH-biotin to hTSHR-BacMPs. (**a**) Saturation binding of bTSH-biotin to hTSHR-BacMPs. Specific binding of bTSH-biotin was calculated by subtracting non-specific binding (a value from wild type AMB-1) from the total binding (a value from hTSHR transformant); (**b**) Competition binding assay. bTSH-biotin (5 μg/mL) was incubated with hTSHR-BacMPs in the presence or absence of excessive non-labeled TSH (500 μg/mL) and assessed for specific TSH binding to hTSHR on BacMPs; (**c**) Scatchard transformation of the binding data from (**a**). The maximum specific binding, *B*_max_ = 6.8 fmol/50 μg BacMPs and dissociation constant, *K*_d_ = 1.9 × 10^−7^ M, were estimated.

The ligand-binding activity of hTSHR on BacMPs was assessed using biotin-labeled bovine TSH (bTSH-biotin) and alkaline phosphatase (AP)-conjugated streptavidin. bTSH-biotin bound TSHR on BacMPs in a dose-dependent manner (Figure 5a), and binding was inhibited in the presence of excessive non-labeled bTSH (Figure 5b). These results indicate that hTSHR on BacMPs has specific ligand-binding activity. Scatchard analysis of the ligand binding assay showed that the maximum specific binding (*B*_max_) value was 6.8 fmol/50 μg of hTSHR-BacMPs, and the dissociation constant (*K*_d_) was calculated to be 1.9 × 10^−7^ M (Figure 5c).

### 2.6. Binding of Autoantibody to hTSHR on BacMPs

**Figure 6:**
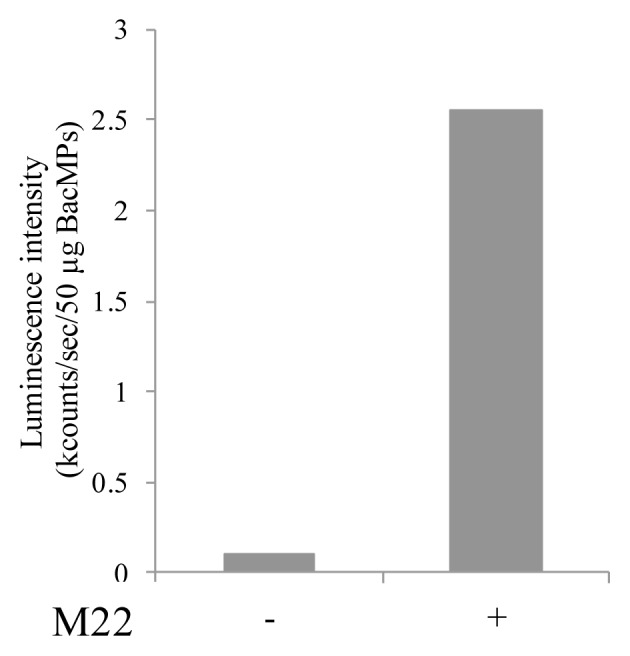
Autoantibody binding assay. Luminescence intensity was calculated by subtracting the value of Mms13-hTSHR transformant from that of wild type. + with autoantibody; − without autoantibody.

To determine whether hTSHR on BacMPs has autoantibody binding activity, the binding of M22 anti-human TSHR autoantibody to hTSHR-BacMPs was evaluated. M22 was detected with AP-conjugated anti-human IgG (Figure 6), and there was no difference in signal intensity with or without M22 antibody in BacMPs extracted from wild-type AMB-1 (data not shown). These results indicated that hTSHR on BacMPs has autoantibody binding activity, although this appears to be weaker than its binding to TSH.

## 3. Discussion

In this study, full-length hTSHR was successfully expressed on BacMPs using a tetracycline-inducible expression system. Most studies to date on TSHR binding to TSH ligand or autoantibodies have relied upon the ectodomain of TSHR due to difficulties associated with expression of full-length protein, especially in prokaryotes. It should be noted however that it is unknown whether all the types of autoantibodies bind equally to both ectodomain and full-length TSHR. Using bacterially-expressed hTSHR ectodomain, some groups have demonstrated ligand binding activity either in a native form [15], or reactivity toward serum autoantibody of a denatured form [16], although not all have been successful [17–19]. Soluble, full-length hTSHR expression has been reported in a prokaryotic system [20], in which immunoreactivity to sera in two GD patients was shown by Western blotting. It has remained unknown, therefore, whether the native form of bacteria-specific hTSHR has ligand or autoantibody binding activity. Here, functional expression of full-length hTSHR on BacMPs has been performed, suggesting that the lipid bilayer on the surface of BacMPs may be suitable for maintaining the functional structure of hTSHR in a membrane-integrated form. Our data suggest that a proportion of the hTSHR molecules are integrated into the BacMPs, although analysis of topological features remains a challenge.

The *K*_d_ value of Mms13-hTSHR determined from our experiment (1.9 × 10^−7^ M) is two orders of magnitude less than that observed with native porcine TSHR (~10^−9^ M) in studies in which the ectodomain or full-length of hTSHR was expressed in mammalian cells or insect cells [21–23], but is relatively close to that of the hTSHR ectodomain expressed in prokaryotes [15]. These observations are consistent with a previous report demonstrating that tyrosine sulfation of TSHR, which is not present in prokaryotes, is important for high-affinity ligand binding [24]. Another possibility is that the *N*-terminal Mms13 protein may have influenced the binding affinity. Moreover, importantly, TSHR is heavily glycosylated, and approximately 40% of the mass of the extracellular TSH binding domain is carbohydrate [25,26]. While the issue of whether glycosylation contributes to TSH- or M22-binding site remains controversial [7], it can be speculated that it is required for correct polypeptide folding [26]. Therefore, the lower binding activity may also be explained by either lack of glycosylation or the resulting partially incorrect folding. Strategies to increase the expression level will include optimization of induction conditions [13], selection of an appropriate promoter [27] and/or genetic modification of the host strain [28,29]. In addition, it may be helpful to test the effect on ligand binding activity of lower growth temperatures, co-expression of molecular chaperones [30] or the formation of disulfide bonds [31]. Regardless, our data are the first report in a bacterial expression system of the production of hTSHR-BacMPs with both ligand binding activity and, to a lesser extent, M22 autoantibody binding activity, and demonstrate the usefulness of BacMPs as a carrier for functional protein expression.

Recently, fully-automated immunoassays for the detection of TRAb have become available and numerous reports have demonstrated high sensitivity and specificity of these assays in the diagnosis of GD [32–34]. Although the low affinity of the BacMP-generated TSHR currently limits its application to GD diagnostics, we believe our unique transmembrane protein expression system has potential as a novel tool for *in vitro* ligand-receptor interaction analysis.

## 4. Experimental Section

### 4.1. Bacterial Cell Strains

*Escherichia coli* strain EC100 (Epicentre, Madison, WI, USA) was used as a host for gene cloning. *E. coli* cells were grown at 37 °C in Luria-Bertani (LB) agar or medium containing 50 μg/mL ampicillin for plasmid selection. *M. magneticum* AMB-1 (ATCC 700264) was microaerobically cultured in magnetic spirillum growth medium (MSGM) at 25 °C as described previously [35]. AMB-1 transformants were cultured under the same conditions with 5 μg/mL ampicillin. To induce hTSHR gene expression, 500 ng/mL anhydrotetracycline (ATc; Cole-Parmer Instrument, Vernon Hills, IL, USA) was added at mid-log phase and further cultured for 72 h.

### 4.2. Vector Construction

To construct Mms13-hTSHR expression vector, full-length human hTSHR cDNA was PCR-amplified with a forward primer 5′-ATGAGGCCGGCGGACTTG-3′ and a reverse primer 5′-TTAGATATCTTTGTCGTCGTCGTCCTTGTAGTCCAAAACCGTTTGCATATACTCTTCTGAG-3′ (containing FLAG-tag sequence) using a plasmid encoding hTSHR cDNA as a template. The PCR fragment was cloned into *Ssp*I-digested pUMtOR13, a derivative of a tetracycline-inducible vector pUMtOR [13], to express mms13-hTSHR fusion gene from tetracycline-inducible msp1 promoter. The construct was designated pUMtOR13TSHR.

To construct Mms13-GFP expression vector, a gene encoding GFP was PCR-amplified with a forward primer 5′-GTGAGCAAGGGCGCCGAG-3′ and a reverse primer 5′-TTACTTGTACAGCTCATC-3′ using pAcGFP1 (TAKARA BIO, Shiga, Japan) as a template. The PCR fragment was cloned into *Ssp*I-digested pUMGP16M13 [11]. The construct was designated pUM13GFP.

### 4.3. Preparation of BacMPs

AMB-1 cells from 5 L culture were collected by centrifugation at 9000 × *g* for 10 min at 4 °C, resuspended in 40 mL of 10 mM HEPES buffer (pH 7.4), and disrupted by a pass through a French press at 1500 kg/cm^2^ (Ohtake Works, Tokyo, Japan). BacMPs extracted from wild type or transformants of AMB-1 were collected from the disrupted cell fraction using a columnar neodymium-boron (Nd-B) magnet and washed 10 times with HEPES buffer. The concentration of BacMPs in suspension was determined by measuring the optical density at 660 nm with a UV-2200 spectrophotometer (Shimadzu, Kyoto, Japan). A value of 1.0 corresponded to 172 μg (dry weight) BacMPs/mL.

### 4.4. Expression Analysis of hTSHR on BacMPs

#### 4.4.1. ELISA

BacMPs from wild type AMB-1 or hTSHR transformant (50 μg each) were mixed with 1 μg/mL of mouse anti-human TSHR (A10) monoclonal antibody (Santa Cruz Biotechnology, CA, USA) in PBS-T and incubated for 30 min at 25 °C with pulsed sonication every 15 min. BacMPs were then magnetically separated and washed with 200 μL of PBS-T with sonication. Then, 1 μg/mL of alkaline-phosphatase (AP)-conjugated streptavidin (AP-SA; Roche Diagnostics, Basel, Switzerland) was added and incubation was continued. After washing, the BacMPs were resuspended in 50 μL of PBS, followed by the addition of 50 μL of Lumi-Phos 530 (Wako Pure Chemical Industries, Osaka, Japan) as a luminescence substrate. After 5 min of incubation, the luminescence intensity was measured using a Lucy-2 luminometer (Aloka, Tokyo, Japan).

#### 4.4.2. Western Blot Analysis

Membrane proteins from BacMPs were extracted from 2.5 mg of BacMPs as described previously [36]. The membrane proteins were mixed with an equal volume of 2× SDS sample buffer (125 mM Tris-HCl (pH 6.8), 10% 2-mercaptoethanol, 4% sodium dodecyl sulfate (SDS), 10% sucrose, and 0.004% bromophenol blue) and denatured at 100 °C for 30 min. The samples were separated by SDS-polyacrylamide gel electrophoresis (PAGE) through 12.5% (*w*/*v*) gel, and then transferred to a polyvinylidene difluoride (PVDF) membrane. FLAG tag was detected with 1 μg/mL of AP-conjugated anti-FLAG M2 monoclonal antibody (Sigma-Aldrich, St. Louis, MO, USA) and subsequent reaction with 1 μg/mL of AP-conjugated goat anti-mouse IgG (Santa Cruz Biotechnology, Santa Cruz, CA, USA) in phosphate buffered saline with 0.05% Tween 20 (PBS-T). BCIP/NBT-Blue (Sigma-Aldrich, St. Louis, MO, USA) was used as an AP substrate for visualization.

### 4.5. Thrombin Cleavage Assay on BacMPs

Thrombin solution (0–100 units/mL) was added to 200 μg of BacMPs from AMB-1 transformants in HEPES buffer and incubated at 25 °C for 2 h. Antibody binding assay was done as described above with 1 μg/mL of mouse anti-hTSHR A10 antibody for hTSHR transformant (Abcam, Cambridge, UK) or 1 μg/mL of goat anti-GFP antibody for GFP transformant (Rockland Immunochemicals, Gilbertsville, PA, USA), followed by 1 μg/mL of AP-conjugated anti-mouse IgG (Abcam, UK) or anti-goat IgG antibody (Santa Cruz Biotechnology, Santa Cruz, CA, USA), respectively.

### 4.6. Ligand and Autoantibody Binding Assay on BacMPs

For ligand-binding assay, 50 μg each of BacMPs from wild type AMB-1 or hTSHR transformant was mixed with serially diluted biotin-labeled bovine TSH (Sigma-Aldrich, St. Louis, MO, USA) (bTSH-biotin) in PBS-T and incubated for 30 min at 25 °C with pulsed sonication every 15 min. Biotin labeling of bovine TSH was performed using EZ-Link Micro Sulfo-NHS-LC-Biotinylation Kit (Thermo Fisher Scientific, Waltham, MA, USA) according to the manufacturer’s instructions. BacMPs were washed with PBS-T, after which 1 U/mL of AP-SA was added. After 30 min of incubation and subsequent washing with PBS-T, Lumi-Phos 530 was added and chemiluminescence was measured by Lucy-2 luminometer.

For TSHR autoantibody-binding assay, 200 μg of BacMPs was mixed with 1 μg/mL of M22 autoantibody (RSR, Cardiff, UK) and incubated for 30 min at 37 °C. Then bound M22 autoantibody was detected with 1 μg/mL of AP-conjugated anti-human IgG antibody (Abcam, UK) as described earlier.

### 4.7. Determination of *K*_d_ Value for the Interaction of TSH and hTSHR

*K*_d_ value for the interaction of TSH and hTSHR was determined by Scatchard analysis of the TSH binding assay as described above. The amount of bound bTSH-biotin was estimated by addition of an excess amount of AP-SA under the assumption that bTSH-biotin bound to AP-SA at a molecular ratio from 1:1 to 1:3, which made no significant difference on the *K*_d_ value of this study (1.9 × 10^−7^ M).

## 5. Conclusions

Full-length, transmembrane hTSHR with ligand- and autoantibody-binding activity was successfully overexpressed on BacMPs of *M. Magneticum* AMB-1 using a tetracycline-inducible system. To our knowledge, this is the first report of such a system. hTSHR-BacMPs has potential application not only as a diagnostic tool of GD, but also in functional analysis of ligand or autoantibody-receptor interactions.
